# Gender Discrimination among Medical Students in Pakistan: A Cross Sectional Survey

**DOI:** 10.12669/pjms.292.3256

**Published:** 2013-04

**Authors:** Ali Madeeh Hashmi, Amra Rehman, Zeeshan Butt, Muhammad Awais Aftab, Aimen Shahid, Sahar Abbas Khan

**Affiliations:** 1 Ali Madeeh Hashmi, MD, Foreign Professor III (HEC), Psychiatry, King Edward Medical University/Mayo Hospital, Lahore, Pakistan.; 2 Amra Rehman, 4th Year MBBS, CMH Lahore Medical College, Lahore, Pakistan.; 3 Zeeshan Butt, MBBS, Resident in Internal Medicine, King Edward Medical University/Mayo Hospital, Lahore, Pakistan.; 4 Muhammad Awais Aftab, MBBS, Graduate, King Edward Medical University/Mayo Hospital, Lahore, Pakistan.; 5 Aimen Shahid, 4th Year MBBS, CMH Lahore Medical College, Lahore, Pakistan.; 6 Sahar Abbas Khan, 4th Year MBBS, CMH Lahore Medical College, Lahore, Pakistan.

**Keywords:** Gender, Gender discrimination, Medical students, Medical education

## Abstract

***Objective: ***To examine the prevalence and magnitude of gender discrimination experienced by undergraduate medical students, and its repercussions on their academic performance and emotional health.

***Methodology: ***A cross sectional study of 500 medical and dental students studying at a private medical college in Lahore, Pakistan.

***Results: ***Majority (78%) of students reported being victims of gender discrimination. Females were the main perpetrators (70.8%).Most common forms were denied opportunities (63%), followed by neglecting students’ needs (44.3%), and unethical talk (43.6%). Most common places of gender discrimination were teachers’ offices (43.7%) and lecture halls (37.2%). Most of the perpetrators were clerical staff (48%) and professors (43%).Gender discrimination did not affect the academic performance of most victims (62.6%). The most common emotional responses were anger (57.6%), frustration (46.7%) and helplessness (40.3%). 52.4% of students said that gender discrimination still continues and the majority (83.3%) did not report the problem to college authorities.

***Conclusions: ***Results demonstrate that gender discrimination is widely prevalent in undergraduate medical education. Females are both the main victims as well as the main perpetrators. In most cases gender discrimination does not affect academic performance but does cause emotional distress.

## INTRODUCTION

Pakistan has traditionally been a male dominant society. This is gradually changing with increasing education and employment of women especially in the medical profession. In Pakistan’s medical colleges, women account for almost sixty five percent of enrolled students with the numbers increasing every year.

Studies have previously been conducted on topics such as bullying of medical students including in Pakistan^1^^-^^3^ but no study has been carried out to assess the experiences of medical students in Pakistan regarding gender discrimination (or sexism).Gender Discrimination refers to gender based behavior, policies and actions that adversely affect work by leading to disparate treatment or creation of an intimidating environment.^4^‘Sexism’ refers to a belief that one sex is superior to the other and that the superior sex has endowments, rights, prerogatives and status greater than those of the inferior sex.^5^ Sexism results in discrimination in all areas of life and acts as a limiting factor in educational, professional, and psychological development.

Medical students being at the bottom of the hierarchy of medical profession are most vulnerable to harassment, discrimination and abuse. Given that medical college plays a vital role in shaping the character and career of a doctor, establishment of a secure, unprejudiced environment is a challenge for all medical colleges.^6^

We hypothesized that gender bias is not only widely experienced by undergraduate medical students in various forms but also affects their professional and personal development. In this study we examined the prevalence of gender discrimination, the main perpetrators of gender bias and the most common form of sexism experienced by the students. Moreover, we tried to assess the magnitude and severity of the problem according to the beliefs of students.

Another important aspect of our study was to examine the repercussions of gender bias in terms of emotional response and academic performance. We have also attempted to determine the main reason for the existence of gender discrimination in medical institutes.

## METHODOLOGY

This cross sectional study was conducted in CMH Lahore Medical College. Students from first to final year of their medical programs (both MBBS and BDS students) were included. A questionnaire was designed to collect information, which was distributed to students during their regular classes in May 2012. Participation was voluntary and confidentiality was assured. The response rate came out to be 86%.

The first part of the questionnaire was for all students in which they were given the option to agree or disagree with various statements regarding experience and observation of gender discrimination. This was followed by a series of yes/no questions about their own experience.

The second part of questionnaire was specifically for those who experienced gender discrimination. It included questions about the most common form of gender discrimination experienced by them and its effect on their academic performances and emotional wellbeing.

Data analysis was done by using Statistical Package for Social Sciences (SPSS) version 16.0. Chi-square test was used to determine the probability values for nominal data collected.

## RESULTS

A total of 500 students participated in the study; majority (64.8%) were females. Mean age of the students was 21.2 ± 1.4 years. Majority of students were from 3^rd^ year (30.4%), followed by 1^st^ year (23.6%), 4^th^ year (19.8%), final year (13.6%), and 2^nd^ year (12.6%).

Majority believed that females are more likely to experience gender discrimination and most males show gender based behavior (71.8% and 63.8% respectively). Half (51%) of the students had observed their friends experiencing gender discrimination.

A vast majority (78%) of students said that they had been victims of gender discrimination. Females were the main perpetrators (70.8%).Most common forms of gender discrimination were denied opportunities (63%), followed by neglecting students’ needs (44.3%), and unethical talk (43.6%). The most common places of gender discrimination were teachers’ offices (43.7%) followed by lecture halls (37.2%). Most of the perpetrators were clerical staff (48%) and professors (43%).

Gender discrimination did not affect the academic performance of most victims (62.6%). The most common emotional response was anger (57.6%), followed by frustration (46.7%) and helplessness (40.3%). About 52.4% of students said that gender discrimination still continues. The majority (83.3%) said that they did not report this problem to college authorities. The main reason for not reporting gender based behavior to college authorities was that it was useless to report such behavior (64%). (Table-I)

**Table-I:** Gender discrimination among medical students

**Variable**	**n/N**	**%age**
Female students are more likely to experience gender discrimination—Agree	359/500	71.8
Males are more likely to show gender-based behavior — Agree	319/500	63.8
Ever seen your friends experiencing gender discrimination — Yes	238/493	48.3
Regarding gender discrimination, what do you think…		
Does not exist	22/493	4.5
Is of no significance	74/493	15.0
Is not a major concern	301/493	61.1
Is a major problem	96/493	19.5
Have you ever experienced gender discrimination — Yes	390/500	78.0
In most cases, you experienced gender discrimination from — Females	276/390	70.8
Has this behavior affected your academic performance — No	244/390	62.6
Forms of gender discrimination that you experienced…		
Denied opportunities	244/389	63.0
Unethical talk	169/388	43.6
Gender-biased grading in class tests	130/388	33.5
Threat to fail in exams	52/388	13.4
Neglect	172/388	44.3
Time denial	63/389	27.8
What was your emotional response to gender discrimination experienced by you…		
Anger	221/384	57.6
Frustration	178/381	46.7
Anxiety	113/378	29.9
Depression	123/379	32.5
Helplessness	153/380	40.3
Does this gender-based behavior still continues — Yes	204/389	52.4
Did you ever report to authorities about gender discrimination — No	325/390	83.3
Reason for not reporting to higher authorities…		
Dealt with the problem yourself	94/315	29.8
It was not a big problem to be concerned about	124/315	39.4
It was useless to report	208/325	64.0

***Comparison of females with males: ***Both males and females were equally victims of gender discrimination (81.2% vs 76.2%, p value 0.20). Both agreed that females are usually the victims and males the perpetrators of gender discrimination (p values 0.19 and 0.30 respectively). Majority of males and females suffered gender discrimination from females (69.2% vs 71.7%, p value 0.61). Most common form of gender discrimination for both males and females was denied opportunities (68.5% and 59.8% respectively). Anger was the most common emotional response to gender discrimination for both males and females (61% and 55.6% respectively). More female students than males were frustrated because of gender based behaviors (50.6% vs 40.1%, p value 0.04), while anxiety as an emotional response was more common in males than in females (36.2% vs 26.2%, p value 0.04). A significantly higher percentage of male victims of gender discrimination dealt with it compared to female victims (36.8% vs 25.8%, p value 0.04).

## DISCUSSION

In this first survey of gender discrimination faced by medical students in Pakistan, over three quarters (78%) of medical and dental students surveyed reported being victims of gender discrimination, with females being the main perpetrators (70.8%).

In a survey from Canada that focused largely on sexual harassment^7^, 46% of the female medical students reported sexual harassment during medical training. In another, large survey from the US^8^, 42% of senior medical students reported having experienced harassment and 84% reported experiencing belittlement during medical school. These students were significantly more likely to be stressed, depressed, and suicidal, to drink alcohol or to binge drink, and were also significantly less likely to be glad with their profession. In a Swedish study^9^ 59% of respondents reported at least one experience of derogatory jokes and comments, 54% of respondents reported at least one experience of gender-related discrimination, and 22% of respondents reported at least one incident of sexual harassment. Women, especially undergraduate women, were more often exposed to all kinds of harassment than were men. In a survey of residents undergoing training in Canada^10^ psychological abuse was reported by 50% of the residents, with discrimination experienced more often by female residents. Sexual harassment was in the form of sexist jokes, flirtation and unwanted compliments on their dress or figure. The most frequent emotional reactions to sexual harassment were embarrassment (reported by 24.0%), anger (by 23.4%) and frustration (20.8%).

The results of our study of medical students are also consistent with a study on prevalence of workplace bullying among junior doctors in Pakistan by Imran N *et al*^11^. 63.8% of them reported being bullied in the last one year. The most frequent perpetrators of bullying were consultants and the majority of victims did not make any official complaints. This suggests that there may not be much difference in the prevalence and manner of discrimination among medical students and junior doctors.

In some studies, the abuse experienced by students can be explained by racial, ethnic or social discrimination^12^, but that is not the case here, since the social, ethnic and racial backgrounds of our students were very homogeneous. It is difficult to generalize but considering similar studies by other authors^13^^,^^14^ mistreatment of medical students seems to be very common. The incidence of gender discrimination in these studies is similar to that reported in our study if all harassing behavior is included. Several studies have documented the long term negative mental health squeal of such abuse including depression, anxiety and alcohol abuse.^13^^,^^15^ In one study on bullying^1^ two protective factors against bullying were a medical college having a policy against bullying and harassment, and perception of availability of support for affected individuals.

One unusual finding in our study was that the majority of the perpetrators were females (70.8%). Most previous studies report females as more likely to be abused or harassed. However, most of these studies have not commented on the gender of the perpetrators. One would expect that males would be the perpetrators in most cases. Therefore, our finding that females were the perpetrators in the majority of cases in our study (70.8%) is unusual and needs to be explored further. One explanation for this could be that as more women move into faculty and administrative positions, the incidence of gender discrimination does not automatically decline i.e. females in authority positions may be just as likely to practice gender discrimination as males. 

Another surprising finding was that gender discrimination did not affect the academic performance of the majority (62.6%) of the victims. One explanation for this may be that since unfortunately discriminatory behavior is ‘institutionalized’ in Pakistan society, its occurrence in a college or university does not cause as much distress as it might in a more egalitarian society. 

The strengths of our study include its reasonably large sample size and a high student response rate. Our study was conducted in one of the private medical colleges in Lahore. A previous study on bullying of medical students^1^ pointed out that its subjects included only 18% of students in private medical colleges. Given the fact that private medical colleges now largely outnumber public institutions, a study of only public colleges may limit generalization of a study’s results.

However, this is also a limitation of our study i.e. that it included only students at one medical college. Further studies need to be conducted using a larger cross section of students from both public and private institutions. Considering the results from previous studies, we can hypothesize, though, that the results would be similar.

Another factor to take into account with regards to our study is that it was conducted in a private medical college managed and run by the Army. One may expect such an institution to have better ethical rules and discipline than a public sector institution, but our study results show that even in an army medical school, majority of students have experienced some form of gender discrimination. As there are no local studies regarding gender discrimination in medical colleges, we are not in a position to compare the results with other institutions.

The common belief about educational institutions is that they are places which are insulated from larger society where students are taught high-level academic skills, and to read, write and do research with an emphasis on equality, dignity, respect and justice. However, as many studies have shown, this is just not the case.^16^ The main tasks of an educational institution – research, teaching and learning – are seriously threatened if members of staff mistreat their students, and this is especially true where women are concerned. As the number of women enrolled in medical colleges continues to rise, this is becoming even more important. The teaching atmosphere in one’s college years is important not only for learning but also for building up a positive professional identity. These attitudes will have a significant impact on graduates' values and behavior in their future working life.

In addition, colleges and universities hire their teachers and researchers from today's students. It is very important to break the ‘cycle of abuse’ and prevent mistreatment from being transmitted to the next generation using different strategies such as ‘role playing’.^17^ Clear and unambiguous policies against harassment and discrimination as well as providing support to affected individuals can be two steps to reduce their incidence.

It is crucial to develop and maintain a supportive atmosphere within medical studies and training to facilitate learning as well as to nurture the personality traits needed to practice as a doctor. Educational intervention and discussion about this is needed for all college staff.

## CONCLUSION

The results of this study demonstrate that gender discrimination is widely prevalent in undergraduate medical education in Pakistan. Females are both the main victims as well as the main perpetrators. In most cases gender discrimination does not affect academic performance but does cause emotional distress. Gender discrimination continues and goes unreported due to the prevalent impression that reporting it to the college authorities is of no consequence.

## Author’s contribution:

Ali Madeeh Hashmi was involved in conception and design of the study, data analysis and interpretation, manuscript writing, and final approval. Amra Rehman contributed to study conception and design, data acquisition, analysis and interpretation, and drafting the article. Zeeshan Butt was involved in data acquisition, statistical analysis and interpretation, along with drafting and editing the manuscript. Muhammad Awais Aftab helped with data interpretation, manuscript writing and final revision. Aimen Shahid and Sahar Abbas Khan contributed to study design, data collection, entry and statistical analysis.
